# Prediabetes and prehypertension in disease free obese adults correlate with an exacerbated systemic proinflammatory milieu

**DOI:** 10.1186/1476-9255-7-36

**Published:** 2010-07-26

**Authors:** Alok K Gupta, William D Johnson

**Affiliations:** 1 Pennington Biomedical Research Center, Louisiana State University System, Baton Rouge, Louisiana, USA

## Abstract

**Background:**

Obesity is a pro-inflammatory state frequently associated with widespread metabolic alterations that include insulin resistance and deregulation of blood pressure (BP). This cascade of events in some measure explains the susceptibility of obese adults for co-morbid conditions like diabetes mellitus and hypertension.

**Hypothesis:**

We hypothesized that an elevated systemic proinflammatory burden correlates with dysglycemia and deregulated blood pressure.

**Methods:**

We analyzed the screening anthropometric and laboratory measures from healthy disease free obese adults (n = 35; women (W) 27, men (M) 8) in a weight loss study.

**Results:**

Healthy obese normoglycemic (fasting serum glucose: FSG <100 mg/dL) women and men compared with healthy obese with prediabetes (FSG 100-125 mg/dL) had no significant differences for age (Mean ± SD: 52 ± 12 vs. 56 ± 9 y), weight (95 ± 11 vs. 99 ± 13 kg), or waist circumference (108 ± 10 vs. 108 ± 11 cm). Normoglycemic group (n = 24; W = 19, M = 5) had normal FSG 92 ± 4 mg/dL, HbA1c 5.4 ± 0.3%, BP 118/75 mm Hg, but had elevated high sensitivity C-reactive protein (hs CRP) 3.7 ± 3 mg/L and fibrinogen 472 ± 76 mg/dL. The group with prediabetes (n = 11; W = 8, M = 3) with significantly higher FSG (106 ± 3 mg/dL; p < 0.0001), HbA1c (5.9 ± 0.5%; p < 0.002), had prehypertension (BP: 127/80 mm Hg) and significantly higher hs CRP (16.9 ± 9 mg/; p < 0.0001) and fibrinogen (599 ± 95 mg/dL; p < 0.0002).

**Conclusions:**

In otherwise healthy disease free obese adults, a higher degree of systemic inflammation is associated with prediabetes and prehypertension.

## Introduction

Overweight and obese adults in comparison to their non-obese counterparts, have a greater susceptibility for subsequently developing diabetes mellitus and/or hypertension [1,2]. Obesity is a recognized pro-inflammatory state prone for broad alterations of the metabolic milieu, which include increased insulin resistance and loss of blood pressure control [3]. The effect of systemic inflammation and insulin resistance upon blood glucose concentration and blood pressure control, early in the course of the developing overweight and obese condition, prior to the onset of overt diabetes mellitus and/or hypertension, is unclear. It is also not transparent whether the pro-inflammatory state determines the insulin resistant condition or insulin resistance causes increased systemic inflammation.

An expanding visceral adipose tissue compartment [4] (clinical correlate: increased waist circumference), is an altered distribution pattern that is believed to impair adipose tissue function and increase cardiovascular disease (CVD) risk [5]. The altered adipose tissue secretions with auto, para and endocrine effects, appear to influence multiple metabolic pathways, including those that modulate glycemia and blood pressure control [6]. This altered adipose tissue [7-9] secretory activity can unhinge the anti-inflammatory and pro-inflammatory balance favoring inflammation, fostering dysglycemia (clinical correlate: prediabetes), and disrupting blood pressure control (clinical correlate: prehypertension). The escalating inflammation can also result in abnormal circadian blood pressure variability and endothelial dysfunction (unpublished observations, currently in review), which over the long term may lead to cardiovascular disease [10].

Prediabetes and prehypertension in healthy overweight or obese adults can thus be early markers of an expanded visceral adipose tissue driven adipose tissue dysfunction (systemic inflammation) prior to the onset of frank diabetes and/or hypertension. In the present study, we hypothesized that an increasing systemic proinflammatory burden correlates with dysglycemia and deregulated blood pressure. We investigated this hypothesis by comparing disease free obese normoglycemic women and men with disease free obese adults with prediabetes.

## Methods

### Study Design

Screening measures from healthy disease free obese subjects (n = 35) screening for a weight loss study at the Outpatient Clinic, Pennington Biomedical Research Center (PBRC) were used for this study. The Pennington Biomedical Research Center is a campus of the Louisiana State University System and conducts basic, clinical and population research. The research enterprise at the Center includes 80 faculty and more than 40 post-doctoral fellows who comprise a network of 57 laboratories supported by lab technicians, nurses, dieticians, and support personnel, and 19 highly specialized core service facilities. The Center's nearly 600 employees occupy several buildings on the 234-acre campus.

### Study subjects

Healthy disease free obese non-smoking men and women between 35-75 years, with no personal history of or ongoing treatment with chronic intake of prescription medications (like for diabetes mellitus, hypertension or other cardaic, renal, gastro-intestinal, pulmonary, or any other systemic disease process). All subjects had read, understood, and signed a PBRC institutional review board approved consent form. The subjects were stratified into two groups: the group with normoglycemia was compared with the group with prediabetes.

### Demographic, Anthropometric and Laboratory measures

Standard demographic and anthropometric measures were obtained for all subjects. Waist circumference (a surrogate marker for central adiposity), serum high sensitivity C reactive protein (hs CRP) and fibrinogen (for assessment of systemic inflammation), fasting serum glucose and HbA1C (for assessing glycemic status) and fasting complete lipid profile (for assessing serum lipid sub-fractions and obtaining cardaic risk ratios) were obtained. Serum uric acid, total white blood cell count (as measures for systemic inflammation), Lp(a), ApoB and ApoA1 (as measures for cardiovascular risk) were also obtained.

### Normoglycemia, prediabetes and prehypertension

Normoglycemia was defined as fasting serum glucose (FSG) less than 100 mg/dL and prediabetes as FSG more than 100 mg/dL but less then 126 mg/dL (Prediabetes: impaired fasting glucose (IFG) *and/or *impaired glucose tolerance (IGT): American Diabetes Association diagnostic criteria [11]). A glycosylated hemoglobin (HbA1C) between 5.7 and 6.4% (recently approved by the ADA) was also used for the diagnosis of prediabetes. Prehypertension was diagnosed based on the mean (of two successive assessments after a 5 minute rest) resting clinic blood pressure (BP) measures for systolic blood pressure (SBP) >120 but <139 *and/or *diastolic blood pressure (DBP) >80 but <89 mm Hg (Prehypertension: Joint National Commission 7 criteria [12]).

### Systemic inflammation, cardiovascular risk evaluation

Systemic inflammation was measured with serum concentrations of high sensitivity C reactive protein (hs CRP: *reference range: 0.0-3.0 mg/L*), fibrinogen (*reference range: 150-450 mg/dL*), uric acid (*reference range: 4.0-8.5 mg/dL*) and total white blood cell count (*reference range: 3.5-11.0 10^3^/μl*). Glycemic status was assessed with FSG (*desirable <100 mg/dL*) and HbA1C (*desirable <5.6%*). A fasting lipid profile was obtained for total cholesterol (total-C: *desirable <200 mg/dL*), triglycerides (TG: *desirable <150 mg.dL*), high-density cholesterol, (HDL-C: *desirable >40 and >50 mg/dL in men and women, respectively*) and low-density cholesterol, (LDL-C: *desirable <130 mg/dL*). Cardaic risk ratios were calculated (average reference range: *total-C to HDL-C of 5 *and *LDL-C to HDL-C of 3*). Serum concentrations of Lp(a) *(reference range: 1-30 mg/dL*), apolipoprotein A (Apo A: *reference range: 110-205 mg/dL*), apolipoprotein B (Apo B: *reference range: 55-105 mg/dL*), the major apoproteins for HDL-C and LDL-C, respectively were also measured.

## Results

Table 1 describes the demographic, anthropometric and laboratory measures for all the otherwise healthy disease free obese adults included in the study (n = 35; 27 women and 8 men). These disease free, predominantly women, were middle aged, obese, and displayed central obesity. They exhibited good glycemic control (FSG: 96 ± 8 mg/dL, HbA1C: 5.6 ± 0.4%), had slight elevation of systolic blood pressure, but had normal diastolic blood pressure and heart rate (BP121 ± 13/77 ± 7 mm Hg, heart rate: 69 ± 9 beats per minute).

**Table 1:** Demographic, anthropometric and laboratory measures

Healthy Disease Free Obese Adults (n = 35)			
Age (y)	54 ± 11	LDL-C (Desirable < 160 mg/dL)	131 ± 38
Gender (F/M)	27/8	HDL-C (mg/dL)	***53 ± 13***
Weight (kg)	***95 ± 12***	HDL-C: M (Desirable > 40 mg/dL)	***41 ± 12***
BMI (kg/m^2^)	***34.8 ± 3.5***	HDL-C: F (Desirable > 50 mg/dL	***57 ± 11***
WC (cm)	***108 ± 10***	Total-C/HDL-C ratio (Desirable < 5)	4.1
WC: M (Desirable < 102 cm)	***110 ± 12***	LDL-C/HDL-C ratio (Desirable < 3))	2.5
WC: F (Desirable <88 cm)	***107 ± 10***	Lp(a) Desirable < 30 mg/dL)	***38 ± 34***
SBP (Desirable < 120 mm Hg)	***121 ± 13***	Apo A (Desirable > 110 mg/dL)	166 ± 28
DBP (Desirable < 80 mm Hg)	77 ± 7	Apo B (Desirable < 105 mg/dL)	***112 ± 24***
FSG (Desirable < 100 mg/dL)	96 ± 8	Hs-CRP (Desirable < 3.0 mg/L)	***7.8 ± 8.3***
HbA1c (Desirable < 5.8%)	5.6 ± 0.4	Fibrinogen (Desirable < 450 mg/dL)	***512 ± 101***
Total-C (Desirable < 200 mg/dL)	***215 ± 41***	Tot WBC (Desirable 3.5-11.0 × 10^3 ^μl	6.7 ± 1.9 × 10^3^
TG (Desirable < 150 mg/dL)	***153 ± 83***	Uric Acid (Desirable 4.0-8.5 mg/dL)	5.4 ± 1.1

Summarized as Mean ± SD.

***Italics ***= outside desirable range

Their fasting lipid profile, on average, included a slightly elevated total-C, TG and LDL-C (215 ± 41,153 ± 83,131 ± 38 mg/dL, respectively) with normal HDL-C (53 ± 13 mg/dL). Their cardaic risk ratios, however, were below the average range (4.2 ± 1.3 and 2.6 ± 1.0, respectively). Their Lp(a) and ApoB levels were slightly above the upper limits of normal (38 ± 34 and 112 ± 24 mg/dL), while the ApoA1 levels were with in normal limits (166 ± 28 mg/dL), attesting to a normal lipoprotein metabolism, with an average cardiovascular disease risk.

They had accentuated systemic pro-inflammatory profiles with high serum hs CRP and fibrinogen concentrations (7.8 ± 8.3 mg/L and 512 ± 101 mg/dL, respectively). Their uric acid and total white blood cell count (5.4 ± 1.1 mg/dL and 6.74 ± 1.7 10^3^/μl) were normal.

Table 2 details the cardiometabolic risk profile in healthy disease free obese subjects with normoglycemia (n = 24; 19 women, 5 men), compared with those with prediabetes (n = 11; 8 women, 3 men). Differences in means between groups (normoglycemia vs. prediabetes) were not significantly different for age (Mean ± SD: 52 ± 12 vs. 56 ± 9 y; range 28-68 and 37-68 y, respectively), weight (95 ± 11 vs. 99 ± 13 kg) or waist circumference (108 ± 10 vs. 108 ± 11 cm). The group with normoglycemia had normal means for FSG (92 ± 4 mg/dL), HbA1c (5.4 ± 0.3%), mean resting BP (118/75 mm Hg), but on average had elevated hs CRP (3.7 ± 3 mg/L) and fibrinogen (472 ± 76 mg/dL). Compared to the group with normoglycemia, the group with prediabetes, however, had significantly higher fasting serum glucose (106 ± 3; p < 0.0001) and HbA1c (5.9 ± 0.5%; p < 0.002). In the fasting lipid profiles, including lipid sub fractions: only Total-C was significantly higher, with no mean group differences in the LDL-C and TG, the decrease in HDL-C or increase in cardiac risk ratios (Total-C/HDL-C and LDL-C/HDL-C) or Lp(a) and Apo lipoproteins (Apo A1, Apo B). The means for total WBC count and uric acid concentrations in the group with prediabetes was slightly higher, when compared to the group with normoglycemia, but the differences did not reach significance.

**Table 2:** Cardiometabolic profile in Healthy Obese Adults

Descriptive	Normoglycemia (n = 24)	Prediabetes (n = 11)	**p-value**^**a**^
Age (y)	52 ± 12	56 ± 9	NS
Gender (F/M)	19/5	8/3	NS
Weight (kg)	95 ± 11	99 ± 13	NS
WC (cm)	108 ± 10	108 ± 11	NS
SBP (Desirable < 120 mm Hg)	118 ± 13	***127 ± 7***	0.06
DBP (Desirable < 80 mm Hg)	75 ± 7	***80 ± 8***	0.07
FSG (Desirable < 100 mg/dL)	92 ± 4	***106 ± 3***	<0.0001
HbA1c (Desirable < 5.8%)	5.4 ± 0.3	***5.9 ± 0.5***	<0.002
Total-C (Desirable < 200 mg/dL)	194 ± 39	***224 ± 40***	<0.04
Hs-CRP (Desirable < 3.0 mg/L)	***3.7 ± 3.0***	***16.9 ± 9.0***	<0.0001
Fibrinogen (Desirable < 450 mg/dL)	***472 ± 76***	***599 ± 95***	<0.0002
Total WBC (Desirable 3.5-11.0 × 10^3 ^μl)	6.6 ± 1.8 × 10	7.1 ± 2.0 × 10	NS
Uric acid (4.0-8.5 mg/dL)	5.3±1.3	5.7±0.8	NS

Summarized as Mean ± SD.

***Italics ***= Outside desirable range

^a^Student's t-test

**Figure 1:**
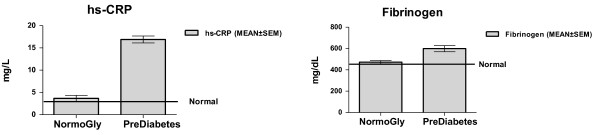
**Systemic Proinflammatory Mileu in Disease Free Normoglycemic and Dysglycemic obese**.

The subjects who had prediabetes, also exhibited prehypertension: mean BP 127/80 mm Hg, along with a significantly higher hs CRP (16.9 ± 9 mg/L; p < 0.0001) and fibrinogen (599 ± 95 mg/dL; p < 0.0002) (Figure 1).

## Discussion

This cross-sectional study evaluated thirty five disease free middle aged, obese women and men with central obesity who were screening for inclusion in a weight loss trial. They all displayed a normal glycemic and lipoprotein metabolism with good blood pressure control, as exhibited by their cardiometabolic risk profile being within the desirable range.

The results from this study confirm that obese subjects subsist in a heightened low-grade systemic inflammatory milieu. Given that overweight and obese subjects exhibit central adiposity (clinical correlate: increased waist circumference), based on a large body of published data [13-17] the low grade systemic inflammation is possibly orchestrated by an expanded visceral adipose tissue compartment. We show that age, weight, waist circumference and cardiometabolic risk profile matched healthy disease free obese subjects with normoglycemia, differ from those with prediabetes only in the grade of systemic inflammation. In the obese state where a basal increase in systemic inflammation is more often shown, it is plausible that at least early in the course of the disorder, prior to the onset of overt diabetes mellitus and hypertension, the grade of inflammation determines the level of glycemia. Thus a higher grade of systemic inflammation is associated with a higher fasting serum glucose concentration. This possibly episodic change in the degree of inflammation, in addition to fostering dysglycemia, appears to deregulate blood pressure control. Subjects with prediabetes thus also tend to have prehypertension.

Obesity is a condition with a multitude of co-morbidities, where an increased mass of dysfunctional adipose tissue in ectopic locations influences the overall total body metabolism with secretions that have auto, para, and endocrine effects [13]. The intra-abdominal or visceral distribution appears to have a significant bearing upon these dysfunctional metabolic modifications, due to its significant association with cardiovascular disease [14]. Macrophage infiltration [15] in the visceral adipose tissue generates hepatic insulin resistance [16] and the association of chronic inflammation with both obesity and chronic diseases [17] suggests that insulin resistance follows inflammation. Elevated systemic markers of inflammation may point towards infiltration of fat in the liver (nonalcoholic fatty liver disease or NAFLD) in the obese state [18], suggesting that inflammation precedes insulin resistance. The vigorous ongoing debate regarding the sequence in which insulin resistance and/or the increase in inflammation develop in the obese [19], however provides a conflicting hypothesis that pro-inflammatory state drives the insulin resistant condition.

Our study confirms findings by others that elevated C reactive protein is associated with glucose levels [20], prediabetes [21], insulin resistance [22] and type 2 diabetes mellitus [23]. It is also in line with the notion that adipose tissue dysfunction drives concurrent metabolic derangements [24] (metabolic syndrome). We extend the literature by showing that early on in this spectrum otherwise healthy disease free obese cardio metabolically neutral subjects with very high levels of hs CRP not only have prediabetes, but also have prehypertension. These results suggest that the degree of systemic inflammation may play a part in the progression of prediabetes to diabetes and prehypertension to hypertension in the obese state.

We postulate that acute exacerbations (clinical correlate: higher total white blood count and uric acid levels in the group with prediabetes: table 2) of an unregulated systemic proinflammatory milieu (clinical correlate: above normal hs CRP and fibrinogen: figure 1) with existing obesity may initially trigger a transient prediabetic and/or prehypertensive condition. A chronic state of stimulated systemic proinflammation in the obese may then perhaps induce an irreversible change in insulin resistance and blood pressure regulation. This may be one of the underlying mechanisms for the development of diabetes mellitus and hypertension in the overweight and obese.

The study has several limitations that warrant discussion. The study subjects were adult asymptomatic volunteers who screened for a weight loss study and may not be representative of the general population. Further, it is a cross-sectional study in which the temporal sequence of emergence of dysregulated assessments is unknown. Finally, the sample size was small so power to detect population differences between the prediabetes and normoglycemia groups may have been compromised. Despite these shortcomings, this investigation documents the finding of a clinical correlation between prediabetes and prehypertension and systemic proinflammation, and establishes a foundation for further investigation of explanatory mechanisms.

In conclusion, in otherwise healthy disease free obese subjects we suggest a link between a higher degree of systemic inflammation and concurrent prediabetes with prehypertension, and postulate a sequence for progression from prediabetes and prehypertension to diabetes and hypertension in obesity. The beneficial effects of a change in diet, an increase in exercise, and securing a weight loss need to be the primary measures for early intervention in this condition.

## Conflict of interests

The authors declare that they have no competing interests.

## Authors' contributions

AKG conceived of the study and drafted the manuscript. WDJ performed the statistical analysis and edited the manuscript. Both authors have read and approved the final manuscript.
